# A Legal Framework for Reducing Violence in Healthcare Institutions: A Comparative Study and Policy Recommendations

**DOI:** 10.1007/s41649-025-00400-1

**Published:** 2026-04-30

**Authors:** Narong Kiettikunwong, Pongmanut Deeod

**Affiliations:** 1 College of Local Administration, Khon Kaen University, Khon Kaen, Thailand; 2 Faculty of Humanities and Social Sciences, Khon Kaen University, Khon Kaen, Thailand

**Keywords:** Healthcare institutions, Violence prevention, Normative legal theory, Comparative health law, Global health governance

## Abstract

Violence against healthcare workers poses a serious global threat to both individual safety and institutional integrity. Yet, many legal systems still rely on general criminal statutes that fail to address the specific vulnerabilities of healthcare settings. This study employs a comparative legal analysis, integrating normative philosophical frameworks—specifically consequentialism and retributivism—to evaluate how seven jurisdictions (Canada, India, Oklahoma–USA, England, Thailand, Bangladesh, and China) respond to healthcare violence. Findings show that jurisdictions with healthcare-specific legislation, such as Canada, China, and England, more effectively align legal sanctions with deterrence, institutional resilience, and moral accountability. In contrast, countries relying solely on general criminal law, such as Thailand and Bangladesh, provide limited normative clarity and protection. Intermediate models, such as India and Oklahoma, offer context-specific responses but lack comprehensive continuity. Synthesizing these insights, the study proposes a unified legal framework grounded in legal specificity, proportionality, and a balanced normative rationale. The model contributes to ethical discourse, informs legal reform, and supports Sustainable Development Goals (SDGs) 3, 8, and 16. By bridging comparative law and moral philosophy, this study provides actionable strategies to safeguard healthcare workers and strengthen justice within health systems.

## Introduction

Despite the enduring ethical mandate to safeguard healthcare institutions as spaces of healing and protection, violence within these environments remains a pervasive global challenge. Article 18 of the Geneva Conventions asserts that even amid war or disaster, hospitals must be sanctuaries free from aggression, declaring that “civilian hospitals organized to give care to the wounded and sick, the infirm and maternity cases, may in no circumstances be the object of attack” (Geneva Convention 1949). Yet, in practice, these protections are routinely violated. The World Health Organization reports that between 8 and 38% of health workers suffer physical violence at some point in their careers, with categories of workers most at risk including nurses and other staff directly involved in patient care, emergency room staff, and paramedics (World Health Organization 2024). Research specifically examining psychiatric settings reveals particularly elevated risks, with healthcare professionals in psychiatric units experiencing approximately three times higher violence rates compared to other healthcare settings and 55% of nurses in psychiatric settings having experienced physical assault (Phillips 2016; Spector et al. 2014). Recent systematic reviews indicate that workplace violence against healthcare professionals is extensive, with overall prevalence reaching 62.4%. This includes verbal abuse (61.2%), psychological violence (50.8%), threats (39.5%), physical violence (13.7%), and sexual harassment (6.3%) (Sari et al. 2023). U.S. Bureau of Labor Statistics data shows that healthcare workers accounted for 73% of all nonfatal workplace injuries and illnesses due to violence in 2018, with healthcare industries experiencing violence rates five times higher than other sectors (U.S. Bureau of Labor Statistics 2020). This epidemic of violence is not isolated to specific regions but reflects a global crisis, as evidenced by Vento et al. (2020) and Liu et al. (2019) which recorded over 1200 violent incidents against healthcare workers across 90 countries within a single year.

Beyond physical harm, such violence exacts significant psychosocial and systemic costs. It deteriorates the mental health of healthcare workers (Ventura-Madangeng and Wilson 2009), diminishes job satisfaction, undermines employee retention (Wilkes et al. 2010), and ultimately compromises patient care. The World Health Organization (2023) notes that healthcare facilities plagued by frequent violence report up to 40% higher staff turnover and 30% lower patient satisfaction, reflecting broader institutional vulnerabilities (Kim et al. 2023). Despite the magnitude of this issue, current legal frameworks remain inadequate. While some jurisdictions have introduced healthcare-specific legislation, most continue to rely on general criminal law, which fails to capture the unique vulnerabilities within healthcare environments (McKay et al. 2020). The International Committee of the Red Cross (2024) reports legal protection gaps in 70% of surveyed countries, particularly regarding penalties and enforcement mechanisms. These gaps present both practical and normative challenges. Excessive punitive measures may escalate violence or deter reporting (Dana 2001), while overly lenient penalties may trivialize offences and erode trust in legal protections (Bibas 2006). While often framed as a legal and policy issue, violence against healthcare workers also raises fundamental bioethical concerns. It undermines the principles of justice, nonmaleficence, and human dignity, and contributes to moral distress and institutional fragility. Addressing such violence therefore requires not only legal deterrence but also ethical justification grounded in normative theory (Beauchamp and Childress 2019).

This study identifies two critical research gaps. First, there is insufficient normative analysis of legal responses to healthcare violence, particularly regarding the philosophical justifications underpinning punishment. While policy debates frequently prioritize deterrence, less attention is given to the moral desert and justice dimensions of legal sanctioning. Second, there remains limited comparative evaluation of how diverse legal systems operationalize these normative principles, leaving policymakers without cohesive models that balance deterrence, proportionality, and moral accountability.

In addressing these gaps, this article pursues three key objectives:

1. To critically analyse existing legal frameworks addressing violence in healthcare institutions through the lens of consequentialist and retributivist theories of punishment
2. To compare legal responses across diverse jurisdictions, identifying both strengths and limitations in current approaches
3. To propose a unified legal framework that balances deterrence and justice while responding to the unique vulnerabilities of healthcare environments, contributing to the advancement of Sustainable Development Goals (SDGs), particularly SDG 3 (Good Health and Well-being), SDG 8 (Decent Work and Economic Growth), and SDG 16 (Peace, Justice, and Strong Institutions)

In pursuing these aims, the article integrates normative theory with comparative policy analysis, offering insights for both philosophical discourse and real-world legal reform. By engaging the ethical foundations of legal punishment alongside practical policy implications, this study fosters a dialogue between philosophy, law, and health policy, advancing scholarly debate and informing institutional strategies to protect healthcare workers and patients alike.

Moreover, this study focuses specifically on unorganized, interpersonal violence that occurs within routine healthcare environments—such as verbal or physical aggression by patients, families, or visitors toward healthcare workers. It deliberately excludes violence in conflict zones, politically motivated or state‑sponsored attacks, and systematic violence against healthcare institutions in war or fragile state contexts. These forms of violence involve distinct geopolitical and humanitarian considerations that fall outside the scope of the present normative‑legal analysis. By delimiting the study to everyday professional interactions in clinical and hospital settings, the analysis ensures conceptual coherence and allows a more precise evaluation of how legal systems address the ethical and institutional dimensions of workplace safety in healthcare.

## Methods

### Research Design

This study employs a comparative legal analysis framework that integrates doctrinal legal research with normative philosophical inquiry and policy evaluation. The approach is informed by recognized methodological foundations in comparative law (Siems 2018; Reimann and Zimmermann 2006), allowing for the structured examination of statutory texts, enforcement mechanisms, and interpretive practices across jurisdictions. It emphasizes not only the functional and institutional aspects of the law but also their normative underpinnings and implications.

A semi-systematic comparative approach was adopted to enhance transparency and replicability, particularly suitable for studies that aim to integrate conceptual synthesis with legal-institutional comparisons (Gough et al. 2012). This involved a clearly defined process of jurisdiction selection, legal text extraction, and interpretive coding, ensuring methodological rigour while accommodating contextual diversity. Jurisdictions were strategically selected—Canada, India, the USA (Oklahoma), England, Thailand Bangladesh, and China—to capture maximum variation in legal traditions (common, mixed, and civil law systems), political regimes (liberal democratic vs. centralized authoritarian), geographic regions, economic development levels, and healthcare system governance, in line with best practices for comparative legal design (Örücü and Nelken 2007), while ensuring analytical feasibility—namely, the availability of accessible legal texts, sufficient policy data, and context-specific relevance for meaningful comparative evaluation.

Importantly, the scope of analysis is confined to interpersonal, non-conflict forms of violence within regular healthcare delivery contexts. It intentionally excludes politically organized violence, conflict-zone aggression, and wartime attacks on healthcare institutions, which represent a different category of violence with distinct normative, humanitarian, and legal implications. By maintaining this clear focus, the study ensures analytical coherence and avoids conflating divergent legal domains. Furthermore, the study further applies normative philosophical lenses—particularly consequentialism and retributivism—to evaluate how legal responses reflect ethical principles such as deterrence, proportionality, and moral accountability. This theoretical integration enables the development of a prescriptive legal framework that not only describes existing legislative responses but also offers normatively grounded recommendations for reform and harmonization.

### Data Collection

Primary sources included constitutional provisions, statutory legislation, regulatory frameworks, and relevant case law from each jurisdiction, accessed through official government repositories and established legal databases. Documents were included if they met three criteria: (1) direct legal relevance to healthcare violence prevention or response; (2) formal adoption by legislative or judicial authority; and (3) public accessibility through official records. Secondary sources encompassed government reports on healthcare violence statistics, peer-reviewed academic literature analysing legal effectiveness, publications from international organizations including WHO and ILO, and professional association position papers. All sources were evaluated for reliability, currency, and relevance, with language barriers addressed through consultation with legal experts and official translations where available. A reference matrix was constructed to catalog the year, jurisdiction, legal instrument type, and thematic content, allowing systematic comparison across cases.

### Data Analysis

The analytical framework operates on three interconnected levels. Structural analysis examines how each jurisdiction organizes its legal response, including institutional arrangements and procedural mechanisms. Substantive analysis focuses on the scope of protection, definitions of prohibited conduct, and penalty structures. Effectiveness assessment evaluates implementation mechanisms and enforcement outcomes where data is available. Both authors independently coded each jurisdiction’s legal materials using these categories, then jointly reviewed coding results to reconcile interpretive differences. Consensus was reached through iterative discussion until agreement was achieved on all conceptual classifications. The normative framework applies both consequentialist analysis (examining deterrence and public health outcomes) and retributivist analysis (focusing on moral condemnation and proportionality principles). Cross-jurisdictional comparison proceeds systematically across all analytical levels, identifying patterns and variations while accounting for contextual differences. This triangulated process—combining document analysis, normative interpretation, and collaborative validation—ensures methodological transparency and theoretical coherence.

### Ethical Considerations

This research adheres to established ethical standards for legal and policy analysis, involving exclusively publicly available documents without human subjects’ participation. All materials are used in accordance with copyright and intellectual property protections, with careful attention to fair use principles and proper attribution. The analysis maintains objectivity without predetermined bias toward particular policy solutions, while acknowledging potential impacts on vulnerable populations including healthcare workers and patients affected by legal framework changes.

### Limitations

Several limitations affect this study’s scope and generalizability. Methodological constraints include inherent difficulties in comparing legal systems operating within different cultural and institutional contexts. Geographic limitations involve concentration in certain world regions with limited representation from Africa, Latin America, and Oceania. Data availability constraints include limited standardized enforcement statistics and insufficient long-term effectiveness data for recently enacted legislation. Temporal considerations reflect point-in-time analysis of evolving legal frameworks. Although the inclusion of China strengthens the conceptual range of the study, it also introduces challenges of interpretation due to potential opacity in enforcement reporting and ideological differences in legal theory and practice. These limitations are addressed through multiple source triangulation, transparent acknowledgment of constraints, and sensitivity to cultural context in interpretation. The study also does not systematically address forms of institutional violence internal to healthcare settings—such as violence against vulnerable patients or intra-hierarchical mistreatment—unless explicitly covered in national legislation. Future research may expand this scope under the broader framework.

## Philosophical Framework: Consequentialism and Retributivism in Legal Sanctions for Healthcare Violence

Understanding the ethical justifications for legal punishment is essential for crafting effective responses to violence within healthcare institutions. Two dominant normative theories—consequentialism and retributivism—offer contrasting yet complementary lenses through which the legitimacy and structure of punishment can be examined. Consequentialism, rooted in the works of Beccaria (1764) and Bentham (1789), asserts that punishment is justified by its outcomes, specifically its capacity to reduce future harm through deterrence, rehabilitation, or societal protection. Under this view, laws addressing healthcare violence should be designed to prevent future incidents, reduce recidivism, and promote institutional safety (Wood 2010; Pilcher et al. 2024). For instance, Canada’s Bill C-3, which imposes enhanced penalties for intimidation or obstruction of healthcare professionals, exemplifies a consequentialist approach aimed at deterring future aggressors and safeguarding public health infrastructure (Government of Canada 2021).

Retributivism, in contrast, grounded in Kantian moral philosophy and further articulated by theorists such as Moore (1987) and Duff (2001), posits that punishment is morally justified when it gives offenders what they deserve, based on the severity of their wrongdoing. This theory emphasizes the intrinsic moral obligation to hold perpetrators accountable, independent of the consequences, reflecting the principle of justice as fairness (Landa 2009; Stern 2024). The Assaults on Emergency Workers (Offences) Act 2018 in England, which imposes higher penalties for assaults against emergency personnel, can be seen as retributivist in that it recognizes the moral gravity of such offences and responds proportionally to the harm inflicted (Crown Prosecution Service 2023).

While consequentialism and retributivism are often portrayed as opposing frameworks, modern legal systems increasingly recognize the limitations of adopting either approach in isolation. Pure consequentialist models risk legitimizing disproportionate punishments if such measures are believed to deter crime, while strict retributivist frameworks may neglect opportunities for rehabilitation or systemic reform (Lewison 2023). In response, hybrid models have emerged, incorporating elements of both theories to balance deterrence, moral accountability, and proportionality. These models often align with restorative justice principles, which emphasize repairing harm, restoring relationships, and reintegrating offenders into society—approaches particularly relevant in healthcare environments where the relational dynamics between patients, staff, and institutions are complex and ongoing (Braithwaite 2002; Pavlacic et al. 2022). In legal responses to healthcare violence, this hybrid orientation is reflected in policies that combine punitive sanctions with preventive measures such as mandatory reporting systems, support services for affected workers, and workplace violence prevention programmes (Sun et al. 2019).

The concept of proportionality—central to both theories but interpreted differently within each—serves as a critical bridge in evaluating legal sanctions. From a retributivist standpoint, proportionality ensures that punishment matches the moral gravity of the offence, while for consequentialists, proportionality calibrates the severity of sanctions to maximize social benefit without causing undue harm (Berman 2021). In contexts like healthcare institutions, where violence may arise from heightened emotional states, systemic stressors, or mental health crises, strict punitive approaches can sometimes escalate conflicts or exacerbate institutional distrust. For example, Bangladesh’s reliance on general criminal provisions without healthcare-specific statutes may reflect a failure to adequately balance these normative principles, leading to inconsistent enforcement and minimal deterrent effects (Dolinko 1997; Pavlacic et al. 2022).

It is important to clarify that this study does not assert that legislative bodies consciously adhere to either retributivist or consequentialist doctrines when drafting sanctions. Rather, the analysis interprets legislative content through these philosophical frameworks to evaluate whether a given provision predominantly reflects moral blameworthiness or utilitarian reasoning. In many cases, legal provisions may be consistent with both traditions. For example, a harsh penalty could serve both to signal the moral unacceptability of the offence (retributivism) and to deter similar conduct (consequentialism). Where such overlaps exist, our interpretation is guided by indicators such as statutory language, legislative intent where available, procedural inflexibility, and the nature of offences categorized. This approach acknowledges that law is not a philosophically pure artifact but a practical tool influenced by multiple, sometimes competing, ethical considerations (Mingers, and Walsham 2010). By making these evaluative lenses explicit, we aim to contribute a more theoretically grounded and policy-relevant understanding of how legal frameworks can and should respond to violence in healthcare settings.

By engaging with these philosophical foundations, this study not only critiques existing legal frameworks but also lays the groundwork for proposing a unified model that synthesizes deterrence, justice, and proportionality. This normative inquiry situates healthcare violence not merely as a legal or policy issue but as an ethical challenge demanding a carefully calibrated response that upholds both individual rights and institutional integrity. Such a framework is essential for fostering safer healthcare environments, advancing the SDGs, and ensuring that legal sanctions are both ethically justified and practically effective.

## Comparative Legal Responses to Prevent Healthcare Violence Across Jurisdictions

A comparative examination of six jurisdictions—Canada, India, the USA (specifically Oklahoma), England, Thailand, and Bangladesh—reveals a spectrum of legal strategies employed to address violence within healthcare settings. Each country’s approach is reflected in its respective legal instruments and demonstrates varying emphases on consequentialist objectives (such as deterring future harm and safeguarding healthcare systems) and retributivist principles (punishing wrongdoing as a matter of justice). These legal strategies also align, to differing degrees, with broader international commitments, particularly the SDGs discussed earlier. In interpreting these frameworks, this study adopts an explicit evaluative logic: where a clause prioritizes deterrence, institutional continuity, or prevention, it is read as consequentialist; where it centres on moral condemnation, desert, or intrinsic culpability, it is read as retributivist. Because legislative texts rarely declare philosophical allegiance, our analysis “lifts the veil” by reading the language, structure, and proportional calibration of sanctions as moral indicators. Where dual readings are possible, we defend the dominant interpretation through contextual evidence and normative reasoning. (Ghosh 2018).

Canada has recently bolstered its legal framework in direct response to rising incidents of threats and aggression toward healthcare workers, particularly heightened by the COVID-19 pandemic. In 2021, the federal government enacted Bill C-3, which amended the Criminal Code to specifically criminalize intimidating healthcare professionals and obstructing access to healthcare facilities (Government of Canada 2021; Canadian Medical Association 2022). This law created a new offence (Section 423.2 of the Criminal Code) for engaging in conduct that instills fear in patients or health providers to impede the provision of care, as well as for blocking entry to hospitals or clinics. The penalties are significant: the offence is hybrid, meaning prosecutors can proceed summarily or by indictment—with convictions carrying up to 10-year imprisonment in serious cases. Additionally, Bill C-3 added any assault or obstruction against healthcare personnel as an explicit aggravating factor in sentencing for other crimes, ensuring judges impose harsher punishments when healthcare services are targeted.

At first glance, the preventive and aggravating features of this statute can be read through both consequentialist and retributivist lenses. A retributivist interpretation might stress that enhanced penalties serve to express moral condemnation toward attacks on caregivers—a symbolic act of giving offenders their deserved censure. However, our reading classifies Bill C‑3 as predominantly consequentialist because the law’s structure and language emphasize the instrumental purpose of deterrence and protection of systemic functioning. The offence targets behaviours—intimidation and obstruction—that threaten institutional operations, not merely moral transgressions, and the preamble and parliamentary debates framed the legislation as necessary to “ensure continuity of healthcare services and protect patients’ access.” These forward‑looking justifications align with consequentialist reasoning, which judges the legitimacy of punishment by its capacity to prevent future harm rather than its intrinsic moral desert (Huigens 2003).

Still, the inclusion of healthcare personnel as a specifically protected category introduces a clear retributivist dimension. This clause functions as a communicative act affirming the moral worth of healthcare work and the social unacceptability of aggression toward it. We defend this dual interpretation on two grounds. First, the proportional escalation of sanctions signals moral denunciation, which satisfies retributive justice by condemning conduct as inherently wrongful. Second, the concurrent preventive scope—targeting intimidation before physical violence occurs—reveals an explicit consequentialist mechanism aimed at averting disruption. In this sense, the law operationalizes a hybrid model wherein deterrence (future-oriented utility) and condemnation (moral expressivism) are mutually reinforcing rather than conceptually opposed. This balanced reading responds to potential alternative interpretations by showing that what might appear as moral symbolism in isolation functions instrumentally within a deterrent framework.

This combination of preventive clauses (targeting intimidation and obstruction) and sentencing aggravation reflects a predominantly consequentialist logic: the law aims to safeguard the functioning of healthcare systems by deterring potential aggressors. Yet, the explicit recognition of healthcare personnel as a protected category also signals a retributivist undertone—society’s moral censure toward violence against those performing vital public service. Thus, Canada’s Bill C-3 illustrates a dual orientation where deterrence (future-oriented) and condemnation (morally expressive) reinforce each other, an overlap that underscores how modern laws operationalize hybrid ethical reasoning. One strength of the Canadian response is its comprehensive scope—covering not only direct violence but also intimidation and interference with healthcare access, thus protecting both individuals and healthcare institutions. A potential limitation, however, is that these measures are very new; their effectiveness will depend on robust enforcement. Early indications suggest Canada’s commitment to protecting medical staff through legal means is strong, reflecting both a moral stance against such violence and an instrumental aim of safeguarding the healthcare system’s integrity (Government of Canada 2025a, 2025b).

India’s legal response to violence in hospitals has evolved, especially in light of high-profile attacks on doctors and nurses. Even before the pandemic, incidents of angry relatives assaulting physicians in India had prompted calls for stronger legal protection, but enforcement under general criminal laws was often inconsistent. The COVID-19 crisis proved a turning point: amid a surge of attacks on healthcare personnel and facilities during 2020, the Indian government enacted the Epidemic Diseases (Amendment) Act, 2020 (initially promulgated as an Ordinance) to specifically address violence against healthcare service personnel in the context of epidemic response (Hegde 2025). This law expansively defines “healthcare service personnel” to include doctors, nurses, paramedics, and other individuals empowered to take measures to prevent disease spread, and it broadly defines “violent acts” to encompass threats, physical harm, obstruction of duties, and damage to property related to healthcare.

Offences under this Act carry stringent penalties: for general assaults or property damage in healthcare settings during an epidemic, offenders face between 3-month and 5-year imprisonment and fines from INR 50,000 up to INR 200,000. If a healthcare worker suffers grievous hurt (serious injury), the punishment increases to 6-month to 7-year imprisonment with fines between INR 100,000 and INR 500,000 (Government of India 2020). Notably, these crimes were made cognizable and non-bailable, meaning police can arrest without a warrant and courts generally cannot readily grant bail, underscoring a retributive zero-tolerance message.

At a surface level, these provisions appear to represent a classic consequentialist approach: the legislation was enacted swiftly during a public health crisis with a utilitarian goal—to deter violence that might obstruct healthcare delivery, ensuring continuity of services during a national emergency. The forward-looking rationale is clear: preventing harm to both personnel and systems, thus maximizing societal benefit (Boddupalli and Francis 2020). Yet, as the reviewer notes, interpretations are rarely unidimensional. While one might read the law’s severity as instrumental deterrence, the rigidity and symbolic framing of non-bailability suggest more than pragmatic calculus.

We interpret the non-bailable status and elevated penalties—especially in a legal culture where bailability is often presumed—as a signal of retributivist influence. Retributivism holds that punishment is morally necessary when individuals violate serious societal norms, and by making these offences categorically non-bailable, the law expresses an unambiguous denunciation of such conduct. One might counter that these penalties still serve deterrence (Manikis and De Santi 2020). However, the law’s language and legislative speeches underscore the “unacceptability” and “grave immorality” of attacking healers—revealing a deeper assertion: that such acts are not just harmful but inherently wrongful.

To defend this dual reading, we point to two aspects. First, the timing and content reflect crisis-driven consequentialist logic, as the government urgently aimed to restore order and public trust in healthcare. Second, the moral tone embedded in the law’s structure—particularly the elimination of discretionary bail and the encompassing definition of violence—goes beyond effectiveness and speaks to justice and societal values. While the same provisions might be read through consequentialist proportionality (i.e., high penalty to prevent escalation), their application with minimal judicial discretion implies a normative judgment of intolerability (Manikis 2022).

This interpretive duality reflects the complexity of India’s legislative design, where utilitarian and moral rationales converge under crisis-driven lawmaking. A strength of the Indian approach is that it directly addresses healthcare-specific vulnerabilities, even covering damage to hospital property and acts of intimidation, thereby extending legal protection to both people and the infrastructure of care (Nair and Zadey 2022). However, a key limitation is the scope: the law was enacted as an amendment to an epidemic diseases statute, so its enhanced protections formally apply in the context of an epidemic or public health crisis. Outside of such conditions, India largely still relies on general criminal provisions (or state-level medical protection laws where they exist), which may result in uneven protection (Nomani and Parveen 2020).

This selective applicability demonstrates how consequentialist urgency can overshadow long-term normative consistency, leaving retributive justice underapplied in non-emergency contexts. In other words, the same moral condemnation implied by non-bailability is not consistently affirmed when healthcare violence occurs outside epidemic periods—exposing a gap between moral signalling and structural follow-through. There have been ongoing calls by medical associations for a dedicated, year-round law to protect healthcare workers nationally, arguing that the consequentialist benefits of deterrence should not be limited to epidemic times. Enforcement remains a challenge as well—despite the law, incidents of hospital violence continue to occur, and successful prosecutions depend on swift investigative action. Still, India’s recent legal reforms mark a significant step by explicitly recognizing and penalizing violence in healthcare settings, blending utilitarian aims of prevention with a retributive stance of denouncing attacks on caregivers (Sai Kumar et al. 2024; Press Information Bureau, Government of India 2022).

In the USA, legal responses to violence against healthcare workers are governed primarily at the state level, leading to a patchwork of protections across jurisdictions. Among these, Oklahoma serves as a representative case for analysis due to its well-established statute specifically addressing violence against emergency healthcare providers, its breadth of coverage across professional roles, and its long-standing legal framework dating back to 1990. While other states such as Rhode Island, Delaware, and Louisiana have enacted similar or more recent statutes—and federal-level proposals have surfaced in Congress—the Oklahoma example offers a consistent and clearly defined legal structure with measurable prosecutorial outcomes, making it an illustrative jurisdiction for normative comparison.

Oklahoma’s law, found in Title 21 of the Oklahoma Statutes, criminalizes assault and battery against “emergency medical care providers” as a distinct felony offence. Under §21–650.4, any person who intentionally and without justifiable cause commits assault, battery, or both upon an on-duty emergency healthcare provider is, upon conviction, guilty of a felony punishable by up to 2-year imprisonment and/or a fine of up to $1000. The statute defines “emergency medical care provider” broadly—encompassing doctors, residents, nurses, nurse’s aides, ambulance attendants and paramedics, technicians, and even hospital security—which reflects a strength of the law: it recognizes that not only physicians, but a wide range of healthcare workers and first responders, face risks of violence (Oklahoma Legislature 2023).

The Oklahoma approach shows a mix of retributivist and consequentialist elements. By elevating an assault on a healthcare worker to a felony, the law embodies a retributive judgment that attacking those who deliver emergency care is especially wrongful and deserving of criminal condemnation beyond what a standard assault might attract. Yet the calibrated severity of punishment—a maximum of 2 years—reveals an implicit consequentialist concern with proportional deterrence: balancing moral disapproval with realistic behavioural correction. While one might interpret this as a modest retributivist signal, the statute’s structure and contextual targeting suggest an instrumental deterrent orientation. The moral condemnation here is present but moderated, reflecting an effort to harmonize justice with practicality.

One limitation of the Oklahoma law is that, despite being on the books for decades (since 1990, with amendments over time), violent incidents still occur, indicating that law alone is not a panacea; effective enforcement and hospital security measures are essential complements. There may also be challenges in application—for instance, prosecutors must prove the victim was an emergency care provider performing duties at the time, and the statute does not cover attacks on healthcare workers outside an emergency context (or those on duty in non-emergency wards). Nevertheless, Oklahoma’s legal response has institutionalized protection for healthcare workers in its criminal law, demonstrating a commitment to both penalize and prevent violence in medical settings (Phillips 2016).

England has explicitly strengthened its laws to address assaults against healthcare staff and other emergency responders, reflecting a clearly normative orientation. The central instrument is the Assaults on Emergency Workers (Offences) Act 2018, which was enacted in response to mounting reports of attacks on National Health Service (NHS) staff, police, and paramedics in the line of duty (UK Parliament 2018). This Act created a specific offence for common assault or battery committed against an “emergency worker” engaged in their duties, and it increased the maximum sentence for such an assault from 6-month to 12-month imprisonment upon summary conviction. Crucially, the definition of “emergency worker” under this law includes not only police, fire, and rescue personnel, but also any person providing NHS healthcare services, including doctors, nurses, and support staff who interact directly with patients.

The moral architecture of the English approach is overtly retributivist: during the passage of the Act, government ministers emphasized that an attack on an emergency service provider is considered an attack on the social order itself—“an attack on the state as well”—warranting punishment more severe than an ordinary assault on a private citizen (Crown Prosecution Service 2025; Weeks and Broughton 2023). This framing underscores a principled moral stance: that those who risk their lives or dedicate themselves to public service (like healing the sick) deserve special protection and that violations of this social contract merit heightened moral censure.

Simultaneously, the law also incorporates consequentialist logic. The increase in maximum penalties is not only symbolic but intended as a deterrent mechanism aimed at reducing the number of violent incidents in hospitals and emergency settings. As a further measure of instrumental prevention, English courts—even prior to the Act—treated the status of a victim as a public servant (such as a nurse or paramedic) as an aggravating factor in sentencing for more serious offences like actual bodily harm. The 2018 Act codified this practice, embedding it more firmly within statutory sentencing guidance and raising the profile of such crimes within the broader legal and public discourse. One of the strengths of England’s legal response is the clarity and communicative function of the statute. It sends an unambiguous public message: abuse or violence toward healthcare workers is unacceptable and will be met with the full force of the law. This clarity can empower NHS institutions to enforce a “zero tolerance” culture, and indeed many hospitals have prominently displayed notices warning that aggression toward staff may lead to arrest and prosecution. Another strength is the inclusive definition of emergency worker, acknowledging that vulnerability to violence extends beyond doctors to nurses, technicians, and even hospital cleaners who deal with patients.

A limitation, however, is that the law alone has not stemmed the tide of violence—thousands of incidents are reported yearly, ranging from verbal threats to physical assaults (Department of Health and Social Care 2018). Enforcement can be challenging; busy hospital environments and the fact that assailants are often distressed patients or family members (sometimes with diminished capacity) mean that not all incidents result in prosecutions or maximum penalties. Moreover, the law is primarily reactive—it penalizes rather than prevents. On its own, it may not be sufficient to deter impulsive or emotionally charged attacks, especially in high-stress healthcare environments. This underscores the need for complementary measures, such as enhanced hospital security, conflict de-escalation training, and mental health crisis protocols. Nonetheless, England’s legislative response exemplifies a hybrid ethical approach: retributive in its moral condemnation and public symbolism, yet consequentialist in its practical objective to deter future harm through enhanced sentencing and visible enforcement (through heightened sentences) (Weeks and Broughton 2023; Parpworth 2022).

Thailand addresses violence in healthcare settings through the general provisions of the Thai Criminal Code, as there is currently no specialized statute offering targeted protection to medical personnel. Incidents of physical harm to doctors, nurses, or hospital staff are prosecuted as ordinary offences—chiefly assault—with the applicable charge depending on the severity of the injuries. For example, an attack causing minor injury would typically be charged under Section 295 of the Criminal Code (causing bodily harm), which carries a penalty of up to 2-year imprisonment or a fine of up to 4000 Baht, or both. If the assault results in serious, “grievous” injury (such as permanent disfigurement or loss of a limb), Section 297 applies, with significantly higher penalties—from a minimum of 6-month up to 10-year imprisonment for the offender (Royal Thai Government Gazette 1956). Even an act of violence that does not cause physical injury but involves force (for instance, a scuffle or attempt to hit someone) is punishable under Section 391, albeit with a relatively light penalty (often a small fine or short detention).

Thus, Thai law nominally punishes nearly all forms of violence in hospitals. However, the law does not distinguish healthcare contexts from general public settings. This absence of a dedicated “medical workplace violence” statute means the legal framework does not recognize the unique vulnerability of healthcare providers, nor the compounded social harm caused by disrupting medical care. Philosophically, this approach reflects a generalized retributivism—the view that any person who commits assault deserves punishment proportional to the harm caused, regardless of the victim’s occupation. It also presumes that consequentialist deterrence is sufficiently achieved through ordinary criminal sanctions, even in high-risk healthcare environments.

One advantage of this generalist approach is its simplicity and formal equality under law: all victims are treated equally, and perpetrators cannot escape liability even if the setting is a hospital. Prosecutors also have some flexibility to escalate charges in aggravated cases. For instance, if a hospital-based assault involves unlawful entry into restricted hospital areas, authorities may pursue charges under Section 364 (trespass on government property) or Section 365, which allows up to 5-year imprisonment if the trespass occurs at night, by a group, or with threats of force (Royal Thai Government Gazette 2020). These provisions can function as de facto legal reinforcements, albeit indirectly, offering some institutional protection.

However, significant limitations remain. The Thai approach lacks a forward-looking, healthcare-specific deterrence strategy. There is no increased penalty simply because the victim is a medical professional performing lifesaving work, nor are there legal obligations for healthcare institutions to adopt protective measures. This creates a philosophical and practical blind spot: an attack in an emergency room is legally treated the same as a barroom altercation, even though the former may delay care for other patients, demoralize staff, and damage public trust in the healthcare system (Wisong et al. 2022). From a retributive standpoint, one might argue that the law fails to express society’s heightened moral disapproval when healers are harmed in the course of fulfilling professional duties. Enforcement in practice may also be inconsistent. Hospital staff in Thailand, like in many countries, may underreport violent incidents due to cultural norms of tolerance, fear of retaliation, or low confidence in legal remedies. Even when reports are made, cases often move slowly through the regular criminal justice system, without fast-tracked procedures or victim protection protocols (Manitkun 2015; Thai PBS 2019).

In sum, Thailand’s reliance on general criminal law provides a baseline of justice but stops short of a proactive, context-sensitive legal response. It lacks both the expressive condemnation of retributivist reasoning and the tailored incentives of consequentialist prevention. As such, it may miss opportunities to both morally signal and practically prevent violence in healthcare settings.

Bangladesh, like Thailand, does not yet have specialized legislation to combat violence in healthcare settings; instead it relies on general criminal laws to prosecute such incidents (Sohag and Eva 2025). The principal legal framework is the venerable Penal Code of 1860, a relic of the colonial era that remains in force. Under this code, an offender who attacks a doctor, nurse, or any other person in a hospital would be charged with standard offences such as “voluntarily causing hurt” or “voluntarily causing grievous hurt,” depending on the outcome of the violence. For instance, if a doctor is struck and sustains a minor injury or pain, the assailant could be convicted under Section 323 of the Penal Code, which generally provides for up to 1 year of imprisonment. If a weapon or dangerous means are used, Section 324 would apply (punishable by up to 3-year imprisonment). In more severe cases—say a doctor is badly injured or disabled—Section 325 (grievous hurt) prescribes a higher penalty, potentially up to 7- or 10-year imprisonment along with fines. These provisions allow Bangladeshi courts to impose significant punishment on those who perpetrate violence in hospitals, reflecting a basic retributivist principle that causing harm to others warrants state punishment commensurate with the injury (Government of Bangladesh 1860).

However, the crucial distinction is that Bangladesh’s laws do not formally distinguish healthcare workers from other victims of assault, nor do they treat violence in medical settings as aggravating circumstances. There is no dedicated law that increases penalties for attacking a doctor on duty or that treats violence in a hospital as an aggravated offence; an assault in a hospital is legally treated no differently than an assault elsewhere (Shahjalal et al. 2021). This reflects a normative stance that general criminal law provides adequate protection across all contexts—a position that rests on the assumption that retributive justice is fulfilled so long as proportional harm is punished, and that consequentialist deterrence operates uniformly regardless of setting.

This philosophical generalism, however, becomes problematic when examined in light of the specific vulnerabilities of healthcare environments. In practice, this gap has been highlighted by repeated incidents of mob violence against physicians in Bangladeshi hospitals, especially following contentious outcomes like patient deaths (Hasan et al. 2024; Sohag and Eva 2025). These events reveal the inadequacy of assuming that ordinary criminal law, crafted in the nineteenth century, is sufficient to manage the emotionally charged and institutionally sensitive context of modern healthcare delivery. They underscore the need for a legal approach that reflects not only the individual harm suffered but also the broader systemic impact of such violence—on medical morale, public trust, and uninterrupted patient care.

One strength of Bangladesh’s approach is that, on paper, offenders can indeed be held accountable under existing law, and the punishments for serious harm are not trivial. Yet the absence of a tailored legal response is widely seen as a weakness. Medical communities and civil society observers have pointed out that the threat of legal consequences under the Penal Code has not been an adequate deterrent—partly because enforcement suffers from procedural delays and lack of prosecutorial zeal, and partly because perpetrators, often fueled by grief or misinformation, may act impulsively without regard for consequences (Kader et al. 2021). Thus, while retributive mechanisms are technically present, their effectiveness in delivering justice or affirming moral condemnation is questionable.

There have been calls to strengthen the legal framework to deter violence against healthcare workers with swift and decisive action taken against those who violate these laws. Such calls implicitly urge a more consequentialist approach: a law that not only punishes after the fact but actively discourages attacks through higher penalties and quicker processes, and that affirms the societal importance of protecting healthcare providers (Shahjalal et al. 2021). Another limitation in Bangladesh is the lack of formal institutional safeguards mandated by law—for example, no legal requirements for hospitals to have security protocols or for law enforcement to station officers at hospitals—leaving the burden largely on overstretched hospital authorities to manage security (Directorate General of Health Services 2024; Sattar 2021). The result is that Bangladesh’s healthcare workers remain vulnerable, and the legal recourse when violence occurs is to invoke an old, general law that was not crafted with hospitals in mind.

On the positive side, the fact that general laws do apply means victims at least have a path to seek redress, and there have been instances where perpetrators of high-profile hospital assaults were arrested and charged under the Penal Code. Nonetheless, as the comparative picture shows, Bangladesh lags behind jurisdictions that have updated their laws to explicitly prioritize the safety of healthcare environments (World Bank 2021). The reliance on general criminal law may appear egalitarian on its surface, but in failing to address the unique operational, emotional, and societal stakes in healthcare violence, it risks both under-deterrence and moral under-expression.

While the preceding analysis compares legal content, it is also important to recognize that the evolution of these frameworks reflects broader societal and institutional contexts. Legislative design in this domain is deeply shaped by factors such as public trust in governance, cultural attitudes toward healthcare professionals, the strength of professional associations, and the maturity of democratic institutions. For instance, Canada’s explicit criminalization of intimidation and obstruction of healthcare workers can be traced to a sociocultural environment that places high normative value on personal safety, public service integrity, and collective well‑being (Nelson et al. 2023). In contrast, countries such as Bangladesh and Thailand operate within more hierarchical governance structures where social deference, limited legislative agility, and resource constraints can impede specialized lawmaking. India’s crisis‑driven legislative action during the COVID‑19 pandemic similarly demonstrates how emergency contexts can accelerate legal reform under heightened public pressure (Gowd et al. 2021). These contextual distinctions do not diminish the normative significance of the laws but rather highlight how cultural, political, and institutional dynamics mediate the translation of moral principles—like deterrence and justice—into statutory form.

Across these six jurisdictions, we see a range of legal responses—from Canada’s and England’s robust, targeted statutes to Thailand’s and Bangladesh’s reliance on general laws—each with its own blend of consequentialist aims and retributivist philosophy. Countries that enacted specific laws to protect healthcare workers often did so after crises or surges in violence, reflecting a consequentialist drive to prevent future incidents by increasing penalties and a retributive intent to condemn such attacks as especially heinous. These laws show strengths in providing clearer protection and potentially deterring misconduct, though they also encounter practical enforcement challenges and must be calibrated to avoid excessive harshness or unintended consequences (Vento et al. 2020; Stutzenberger and Fisher 2014). On the other hand, jurisdictions without bespoke healthcare violence laws maintain a formal egalitarian approach to punishment that may appear normatively neutral but in fact neglects the distinctive social and moral stakes of violence in medical settings. This generalist model risks underestimating both the instrumental need for stronger deterrence and the expressive function of law in condemning assaults on care providers (Phillips 2016; Gillespie et al. 2010). As such, even if retributive proportionality is preserved in theory, justice may still be undermined by the failure to recognize the relational and institutional context of the violence.

This comparative analysis sets the stage for the following section’s in-depth normative discussion and synthesis, which will evaluate how well these varying approaches fulfill principles of justice and utility. The next section will explore, in a more prescriptive vein, how consequentialism and retributivism should be balanced in the context of healthcare violence, and what an optimal legal framework might entail, not merely by aggregating best practices but by reasoning from underlying ethical commitments and policy trade-offs. Drawing on the strengths and addressing the gaps identified in each jurisdiction’s response, the discussion aims to clarify what healthcare-specific criminal law ought to achieve.

China has implemented a robust, top-down legal framework to combat violence and disorder in hospitals. A pivotal development was the Ninth Amendment in 2015 to the Criminal Law of the PRC, which revised Article 290. This change explicitly criminalized “gathering to disturb social order” in ways that disrupt essential services including medical services (Yang 2015; China Law Translate 2015). In effect, organized hospital disturbances—commonly known in China as “yi nao” (医闹) or “medical disturbances”—are now clearly punishable (Lu et al. 2020). Under the amended Article 290 (1), if a crowd causes serious disruption such that healthcare work cannot proceed and serious losses result, the principal instigators face 3–7-year imprisonment, with other active participants subject to up to 3-year detention or other penalties (China Law Translate 2015). This marked the formal inclusion of *yi nao* in the criminal law, signalling a zero-tolerance stance by the state. Chinese authorities touted this amendment as a means to deter lawless individuals and guide patients toward lawful dispute resolution, thereby protecting medical staff and patients (Lu et al. 2020). The law carries a strong symbolic‑moral message: by enshrining hospital order in criminal statute, the state affirmed that violence or mass harassment in healthcare settings is categorically unacceptable (Lu et al. 2020; Shi et al. 2025).

Beyond the Criminal Law, China deployed targeted policies and enforcement campaigns to maintain hospital security. The National Health Commission (formerly the health ministry), in conjunction with public security organs, launched the “Safe Hospital” initiative in 2015 (Xu et al. 2021). This programme aimed to create safe work environments in health facilities by improving security infrastructure and ensuring rapid police responsiveness (Zhang et al. 2021). For example, many large hospitals established on-site police offices at entrances (part of the “Safe Hospital police” scheme) and installed screening systems to prevent weapons from entering (He et al. 2023). In 2015 and 2016, the government issued joint notices and special action plans to crack down on medical-related crimes and maintain medical order. Public security bureaus were instructed to implement “six measures” to protect medical institutions, ranging from heightened patrols to fast-track prosecution of offenders (Lu et al 2020).

Additional regulations address dispute mediation: a 2018 State Council Regulation on the Prevention and Handling of Medical Disputes provided structured channels for patients to air grievances legally, aiming to defuse conflicts before they escalate (Shi et al. 2025). Recent laws have reinforced these efforts—for instance, the 2021 Physicians Law and 2019 Basic Healthcare and Health Promotion Law include provisions on protecting healthcare personnel and penalizing interference with medical services (Zhao 2021). China has even leveraged its emerging social credit system: a 2018 memorandum created joint disciplinary blacklists for individuals who seriously endanger normal medical order, thereby imposing civil penalties (like travel or loan restrictions) in addition to criminal ones (Lu et al. 2020).

Enforcement in China has a pronounced top-down character. Authorities regularly conduct sweep campaigns against *yi nao* syndicates (groups that organize paid protests or violence in hospitals) and swiftly prosecute high-profile cases to set examples. The emphasis is on deterrence and rapid repression of any incident: police are stationed in many hospitals and will arrest perpetrators on-site, reflecting the state’s resolve to “maintain normal medical order as the ‘last line of defense’” (Lu et al. 2020). This top-down approach appears to have had some practical effectiveness. Incidents of major hospital riots or organized disturbances have reportedly declined since the mid-2010s, and one cross-country analysis noted that the rate of reported violence against health workers in China is significantly lower than in India (which lacks such stringent enforcement) (Zhang et al. 2021). However, questions remain about underreporting and the heavy-handed use of public order laws. From a normative perspective, China’s legal response carries a strong retributivist signal—it condemns attacks on healthcare as an affront to public order and justice, meriting strict punishment—while also pursuing consequentialist goals of preventing harm (through deterrence and security measures). We discuss these normative dimensions in detail below.

## Discussion and Normative Synthesis: Toward a Unified Legal Framework

The comparative analysis of legal responses to violence in healthcare institutions across six jurisdictions reveals a landscape marked by considerable diversity in both normative orientation and practical implementation. Countries such as Canada, England, and China exemplify targeted legislative interventions that embody a blend of consequentialist deterrence and retributivist moral condemnation, while Thailand and Bangladesh remain anchored in general criminal law, which lacks the specificity and normative signalling needed to address the distinct vulnerabilities of healthcare environments. India and the USA (Oklahoma) represent intermediate models that combine limited specific statutes with general provisions, often triggered during crises. This variation underscores a fundamental question: what should constitute an ethically justified and practically effective legal response to healthcare violence?

At its core, the legal sanctioning of healthcare violence must satisfy consequentialist aims by preventing future harm, protecting the healthcare workforce, and maintaining public trust in healthcare institutions (Kavanagh et al. 2024). The Canadian model, particularly through Bill C-3, illustrates this well by criminalizing not only physical assaults but also intimidation and obstruction (Department of Justice Canada 2021; Duong and Vogel 2022), thus recognizing that disruptions to healthcare provision—even absent physical violence—can jeopardize the broader functioning of health systems. This approach aligns with SDG 3 (Good Health and Well-being) by ensuring uninterrupted healthcare delivery and with SDG 16 (Peace, Justice, and Strong Institutions) by reinforcing the authority and safety of healthcare environments. However, Canada’s framework also incorporates retributive elements, such as enhanced sentencing for offenders who target healthcare workers, reflecting a societal judgment that such acts deserve heightened moral censure (Centre for International Sustainable Development Law 2020).

Similarly, China’s approach, though grounded in a civil law tradition and a centrally administered governance model, reinforces strong deterrent mechanisms by explicitly criminalizing violence against medical personnel under Article 290 of the Criminal Law and supplementary judicial interpretations. The state’s firm penal response to “yi nao” (medical disturbances) reflects an instrumental logic of public order preservation, coupled with a moral condemnation narrative that frames attacks on healthcare workers as threats to national harmony and social stability (Lee 2019). While the philosophical framing may differ—leaning more toward utilitarian collective interest than liberal individual rights—the outcome parallels consequentialist and retributivist logics by imposing severe punishments to deter and denounce such acts. However, critics argue that China’s model may emphasize compliance over procedural fairness, raising questions about the proportionality of its criminal justice response and the autonomy of judicial reasoning (Lu et al. 2020).

In contrast, England’s Assaults on Emergency Workers (Offences) Act 2018 explicitly anchors its justification in the retributivist claim that assaults on healthcare staff undermine the social order, warranting harsher penalties (UK Parliament 2018). Yet, these laws also serve consequentialist objectives by raising the perceived costs of such offences, aiming to deter would-be aggressors (Weeks and Broughton 2023; Murray et al. 2020). Jurisdictions like Thailand and Bangladesh, which rely solely on general criminal statutes, fail to capture the unique moral and institutional stakes involved in healthcare violence. Their reliance on standard assault provisions, without heightened penalties or special legal recognition for healthcare contexts, reflects a formal egalitarianism that treats all assaults equally, irrespective of setting or victim (Suwittawat 2022; Kader et al. 2021). This approach aligns with a narrow retributivism, where punishment correlates strictly with the degree of harm caused. Yet, this fails to recognize that violence in healthcare settings carries multifaceted harms—it not only injures individuals but also disrupts vital services, erodes workforce morale, and diminishes public trust in healthcare institutions. Such an omission neglects consequentialist concerns and overlooks proportionality in a broader sense, where the social impact of the crime should inform the severity of the response (Kabir et al. 2022; Nithimathachoke and Wichiennopparat 2021; Pummala, et al. 2023).

The USA (Oklahoma) and India represent intermediate models. Oklahoma’s law provides felony-level protection for emergency healthcare workers, reinforcing both deterrence and moral accountability, though the penalties remain moderate and confined to specific roles and contexts (Phillips 2016; Wolf et al. 2020). India’s Epidemic Diseases (Amendment) Act, 2020 demonstrates an effective consequentialist response during public health emergencies, incorporating severe penalties and non-bailable offences to ensure compliance and protect healthcare workers during crises (Hegde 2025; Nair and Zadey 2022). However, its limitation to epidemic contexts leaves healthcare personnel vulnerable outside such periods, exposing a gap in consistency. These cases highlight the importance of flexibility within legal frameworks: laws must adapt to heightened risks in emergencies while maintaining baseline protections at all times.

Synthesizing these findings, this study proposes a unified legal framework characterized by three core principles: specificity, proportionality, and integration of deterrence with moral accountability. First, specificity demands that legal systems recognize healthcare violence as a distinct category of offence, incorporating provisions that address both physical assaults and institutional disruptions (e.g., intimidation, vandalism) (Kwon et al. 2019; Sari et al. 2023). This recognition reflects not only consequentialist priorities—ensuring operational continuity and safety—but also retributivist justice, affirming that attacks on healers and the healthcare system are morally reprehensible (Hills 2018). Second, proportionality must guide sentencing, ensuring that penalties reflect both the harm inflicted and the social context of the offence (Bagaric 2000; Brown Coverdale 2025). For example, physical assaults on healthcare workers should warrant higher penalties than similar offences in other settings, due to the compounded harms they cause—personal injury and systemic disruption. However, proportionality also requires calibrating punishment to avoid excessive harshness, particularly in cases involving distressed patients or those with diminished capacity, integrating a restorative justice dimension where appropriate (Pemberton et al. 2007).

Finally, the integration of deterrence and moral accountability calls for a hybrid normative model, where consequentialist and retributivist objectives are balanced. This balance ensures that laws are not only instrumentally effective but also ethically justified, capable of deterring future harm while respecting the dignity and moral agency of offenders (Hills 2018; Bagaric 2000). However, current legislative efforts across jurisdictions tend to narrowly focus on external threats to healthcare workers, often overlooking broader patterns of violence within healthcare institutions. A more inclusive normative framework would also consider structural and interpersonal violence directed at vulnerable patients (e.g., obstetric violence) and at underpowered members of the healthcare workforce (e.g., interns, junior nurses, or minority groups). Among the jurisdictions reviewed, there was little explicit statutory attention to these forms of internal violence. Recognizing and addressing such dynamics could enhance justice and protection across all levels of the healthcare system, and we suggest this as an important area for future legislative development. This framework supports SDG 8 (Decent Work and Economic Growth) by fostering safer workplaces for healthcare professionals and SDG 16 by strengthening institutional justice and public trust (Khan 2016; Hope 2020).

In conclusion, this normative synthesis affirms that no single theoretical lens suffices in isolation. Rather, an integrated legal framework—grounded in specificity, proportionality, and normative balance—provides the most ethically sound and practically effective approach to addressing healthcare violence. Such a model not only protects healthcare workers but also upholds the integrity of healthcare institutions, contributing to global efforts toward sustainable health systems and just societies. Table 1 below synthesizes the normative and policy-relevant dimensions of each jurisdiction’s legal approach, summarizing their respective strengths, limitations, and potential contributions to a unified legal framework.

**Table 1:** Comparative Summary of Legal Responses to Healthcare Violence

Jurisdiction	Retributivist aspects	Consequentialist aspects	Strengths	Weaknesses	Legislative inspiration for unified framework
Canada	Enhanced sentencing reflects moral censure of attacks on healthcare workers	Criminalizes not only assault but also obstruction and intimidation	Specific legislation; dual normative grounding	Limited public awareness of legal changes	Broad scope of protection; integrates symbolic and instrumental norms
India	Strong punitive response during public health emergencies	Severe penalties and rapid enforcement during crises	Effective in crisis; visible political support	Limited to epidemics; lacks continuity	Crisis-based flexibility; scope for extension to normal periods
USA (Oklahoma)	Felony status conveys moral seriousness	Provides legal deterrent in emergency care contexts	Long-standing statute; measurable enforcement	Role-specific and emergency-limited	Institutional clarity; sustained application record
England	Explicitly justifies heightened penalties based on moral order	Law raises perceived cost of offences to deter aggressors	Clear legal signal; embedded in broader justice system reform	Limited enforcement data	Strong retributivist narrative; legislative clarity
Thailand	General assault law reflects equal moral condemnation	Indirect deterrence through general provisions	Universality of legal treatment	No sector-specific recognition; lacks symbolic value	Legal foundation present; needs specification and signalling
Bangladesh	Relies on general criminal law with retributive parity	Assumes general deterrent through existing laws	Baseline protection in legal code	Ignores context-specific harms; underenforced	Requires tailored provisions and enforcement guidance
China	Strong state-backed condemnation of “*yi nao*” behaviour; criminal classification signals societal disapproval	Centralized enforcement deters collective violence; rapid penalties	Demonstrated reduction in hospital violence; top-down efficacy	Authoritarian context limits individual rights; overbroad implementation	Model for enforcement efficacy and administrative coordination

## Conclusion

Violence within healthcare institutions presents a multifaceted ethical, legal, and policy challenge that extends beyond individual acts of harm to encompass broader systemic implications for healthcare delivery, workforce stability, and public trust. This study has examined the legal responses of seven jurisdictions—Canada, India, the USA (Oklahoma), England, Thailand, Bangladesh, and China—through a normative lens grounded in consequentialism, retributivism, and proportionality. The analysis revealed a spectrum of approaches, ranging from robust and targeted statutes that explicitly recognize healthcare violence as a distinct social harm to more generalized legal provisions that inadequately address the specific vulnerabilities of healthcare environments.

The normative evaluation highlighted that effective legal responses must balance deterrence and moral accountability, ensuring that laws not only prevent future violence but also express the moral gravity of attacks on healthcare workers and institutions (Hills 2018; Bagaric 2000). Jurisdictions like Canada and England demonstrate stronger alignment with this balance by integrating enhanced penalties with clear recognition of the unique role of healthcare professionals (Weeks and Broughton 2023; Wolf et al. 2020). Meanwhile, China’s centralized enforcement model demonstrates how deterrence can be powerfully realized through state capacity and legal codification, though it raises normative questions concerning proportionality and procedural fairness (Lu et al. 2020). In contrast, the reliance on general criminal law in Thailand and Bangladesh, while offering baseline protections, falls short of addressing the broader institutional harms and moral dimensions associated with healthcare violence (Shahjalal et al. 2021; Wisong et al. 2022).

Building on these insights, this article has proposed a unified legal framework grounded in specificity, proportionality, and the integration of normative theories, aimed at fostering safer healthcare environments while upholding justice. Such a framework is not only ethically justified but also instrumental in advancing global policy objectives, particularly SDG 3 (Good Health and Well-being), 8 (Decent Work and Economic Growth), and 16 (Peace, Justice, and Strong Institutions) (Khan 2016; Hope 2020). By positioning healthcare violence as both a legal and ethical issue, this study contributes to the ongoing dialogue between philosophy, law, and health policy, offering actionable recommendations for legal reform that transcend jurisdictional boundaries.

Future research should explore the empirical effectiveness of such frameworks, examining their impact on reducing incidents of healthcare violence, improving staff well-being, and enhancing institutional trust. Further normative inquiry is also warranted to assess how emerging legal models might adapt to evolving healthcare challenges, including pandemics, resource shortages, and global health governance reforms. Through such interdisciplinary engagement, legal systems can better fulfill their role in safeguarding healthcare institutions as sanctuaries of healing and justice.

## Data Availability

No new data were created or analyzed in this study.
